# The effect of a low versus high sodium diet on blood pressure in diabetic patients: A systematic review and meta‐analysis of clinical trials

**DOI:** 10.1002/fsn3.3212

**Published:** 2023-01-04

**Authors:** Mahsa Gholizadeh‐Moghaddam, Farnaz Shahdadian, Fatemeh Shirani, Amir Hadi, Cain C. T. Clark, Mohammad Hossein Rouhani

**Affiliations:** ^1^ Nutrition and Food Security Research Center Department of Community Nutrition School of Nutrition and Food Science Isfahan University of Medical Sciences Isfahan Iran; ^2^ Nutrition and Food Security Research Center Department of Clinical Nutrition School of Nutrition and Food Science Isfahan University of Medical Sciences Isfahan Iran; ^3^ Isfahan Endocrine and Metabolism Research Center Isfahan University of Medical Sciences Isfahan Iran; ^4^ Halal Research Center of IRI, Food and Drug Administration Ministry of Health and Medical Education Tehran Iran; ^5^ Centre for Intelligent Healthcare Coventry University Coventry UK

**Keywords:** blood pressure, diabetes, meta‐analysis, sodium, systematic review

## Abstract

There have been numerous clinical trials that have investigated the effect of sodium intake on blood pressure in diabetic patients. The purpose of this systematic review and meta‐analysis was to evaluate the clinical trial studies performed on the effect of low sodium diet (LSD) versus high sodium diet (HSD) on blood pressure in diabetic patients. PubMed, Scopus, and Web of Science were systematically searched from database inception to July 10, 2021. Both type 1 and 2 diabetes was considered. Overall, there were 15 studies included in this meta‐analysis. The weighted (WMD) mean difference with 95% confidence interval (CI) was calculated using a random‐effects model. Risk of bias in the studies was assessed based on the Cochrane collaboration tool and the quality of all the studies was considered as good. Overall, LSD significantly reduced SBP (systolic blood pressure) (WMD: −3.79 mmHg, 95% CI: −6.02, −1.56) and DBP (diastolic blood pressure) (WMD: −1.62 mmHg, 95% CI: −2.84, −0.40), in comparison with HSD, in diabetics. However, LSD had no significant effect on MAP (mean arterial pressure) in comparison with HSD (WMD: −1.81, 95%CI: −5.49, 1.87). Although subgroup analysis could not attenuate heterogeneity in SBP, subgroup analysis in DBP based on duration (≤1 week: WMD: −2.35, 95%CI: −3.69, −1.00, *I* ^*2*^ = 48.9%, *p* = 0.081, >1 week: WMD: −1.04, 95% CI: −2.83, 0.76, *I* ^*2*^ = 74.7%, *p* = 0.003) and study design (cross‐over: WMD: −1.94, 95% CI: −2.71, −1.17, *I* ^*2*^ = 32.1%, *p* = 0.183, parallel: WMD: −2.17, 95% CI: −6.48, 2.13, *I* ^*2*^ = 82.4%, *p* = 0.001) successfully detected sources of heterogeneity. LSD significantly reduced SBP and DBP, however, had no effect on MAP, in comparison with HSD.

## Graphical abstract

This meta‐analysis has examined clinical trial studies conducted in the field of investigating the effect of a low‐sodium diet versus a high‐sodium diet on blood pressure in diabetics. And the result of this meta‐analysis has shown that a low sodium diet significantly reduces systolic and diastolic blood pressure, but has no effect on MAP.

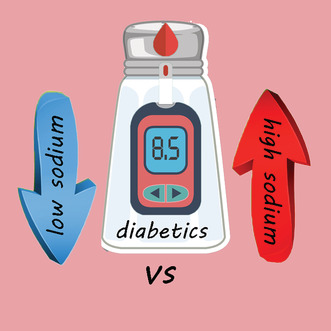

## 1 INTRODUCTION

Diabetes is a chronic disease induced by insulin resistance or reduced insulin secretion. Diabetes mellitus increases the risk of hypertension, even without kidney failure, and the average exchangeable sodium is 10% higher in diabetics relative to non‐diabetics (Gerdts et al., 1996). Approximately 463 million adults are currently living with diabetes worldwide (International Diabetes Federation, 2017); moreover, it is expected that the global prevalence of diabetes will increase several times in the next 20 years (International Diabetes Federation, 2017). High blood pressure (systolic blood pressure (SBP) ≥ 140 and or diastolic blood pressure (DBP) ≥ 80) represents a major comorbidity in patients with diabetes (Passarella et al., 2018; Petrie et al., 2018), where around 74% of adult patients with diabetes have an elevated blood pressure. Hypertension in diabetic patients is one of the main risk factors for diabetes‐associated vascular complications and cardiovascular events (Passarella et al., 2018; Petrie et al., 2018; Saeedi et al., 2019). Also, insulin resistance, hyperglycemia, and an activated sympathetic nervous system play important roles in the pathogenesis of hypertension in patients with type 2 diabetes (Adler et al., 2000; Ohishi, 2018; Vedovato et al., 2004).

There is increasing evidence to advocate that salt sensitivity may be a leading cause of hypertension in diabetic patients (Passarella et al., 2018; Petrie et al., 2018; Vedovato et al., 2004). Indeed, studies have revealed that dietary salt intake could directly cause to high blood pressure. Excessive sodium intake (more than >5 g sodium/day) increases blood pressure and also increases cardiovascular morbidity and mortality (Ferguson et al., 2019; Grillo et al., 2019; Hyseni et al., 2017). A cohort study conducted in people with type 2 diabetes between the ages of 40 to 70 years showed that the higher sodium intake is related to higher risk of CVD, moreover patients with higher HbA1C and intake of sodium were at an elevated risk for CVD (Horikawa et al., 2014). A visually based dietary intervention concluded that reducing the salt intake in short term resulted the SBP improvement in the intervention group compared with the controls (Yokokawa et al., 2020). A 12‐week randomized double‐blind trial demonstrated that a moderate reduction in salt intake resulted in a significant reduction in blood pressure and urinary albumin excretion in diabetic patients (Suckling et al., 2016). The aforementioned study included 46 untreated hypertensive participants with controlled type 2 diabetes or impaired glucose tolerance, and during 2 weeks, the participants were subjected to a low sodium diet, then for 12 weeks, the subjects received six tablets containing 10 mmol of salt or placebo daily. Accordingly, the average urinary sodium in the sodium group was significantly higher than the placebo group, whilst SBP and DBP were significantly higher in the intervention group. On the other hand, a recent observational study in patients with type 2 diabetes revealed that lower salt intake was paradoxically related to an increase in cardiovascular mortality; this observational study was conducted on people with type 2 diabetes who were followed for at least 3 months in terms of urinary albumin excretion. Participants were given general dietary advice, but no detailed assessment of their dietary salt was undertaken. This study showed that, by increasing the 24‐hour urinary sodium (24hUNa) excretion, the all‐cause mortality rate decreased by 28%. After adjusting for competing risk of non‐cardiovascular death and other predictors, 24hUNa was also significantly associated with cardiovascular death (Ekinci et al., 2011). Further, some studies have found that sodium reduction may have adverse effects on diabetes (He et al., 2013; Patel et al., 2015; Thomas et al., 2011). Thus, the role of salt reduction in diabetes mellitus is equivocal, whilst review studies pertaining to the effects of Low Sodium Diet (LSD) versus High Sodium Diet (HSD) on blood pressure in diabetic patients are limited. Hence, we sought to conduct a systematic review and meta‐analysis of a randomized controlled trial (RCTs) to evaluate the effects of low‐sodium diet versus high‐sodium diet on blood pressure in diabetic patients.

## 2 MATERIALS AND METHODS

### 2.1 Search strategy

This study was conducted in accord with the Preferred Reporting Items for Systematic reviews and Meta‐Analyses (PRISMA) statement (Picot et al., 2012). To find relevant articles, a comprehensive electronic search was accomplished on PubMed, ISI Web of Science, and Scopus databases, from database inception up to July 2021. There were no restrictions on the language and time of publication. The search was performed using following keywords: (“sodium”[tiab] OR “salt”[tiab] OR “NaCl”[tiab] OR “Sodium Chloride”[tiab] OR “Sodium restriction”[tiab] OR “Sodium Restricted”[tiab] OR “high‐sodium”[tiab] OR “low‐sodium”[tiab] OR “Diet, Sodium‐Restricted”[mesh] OR “Sodium Chloride”[Mesh] OR “Diet, Sodium‐Restricted”[Mesh]) AND (“blood pressure”[tiab] OR “hypertension”[tiab] OR “hypertensive”[tiab] OR “hypotension”[tiab] “hypotensive”[tiab] OR “systolic blood pressure”[tiab] OR “diastolic blood pressure”[tiab] OR “SBP”[tiab] OR “DBP”[tiab] OR “Blood Pressure”[Mesh] OR “Hypertension”[Mesh] OR “Hypotension”[Mesh]) AND (“Diabetes”[tiab] OR “Diabetic”[tiab] OR “Hyperglycemia”[tiab] OR “Hyperglycemic”[tiab] OR “DM”[tiab] OR “T2DM”[tiab] OR “NIDDM”[tiab] OR “insulin resistance”[tiab] OR “glucose intolerance”[tiab] OR “Diabetes Mellitus”[Mesh] OR “Diabetes Mellitus, Type 1”[Mesh] OR “Hyperglycemia”[Mesh] OR “Diabetes, Gestational”[Mesh] OR “Diabetes Mellitus, Type 2”[Mesh] OR “Insulin Resistance”[Mesh] OR “glucose intolerance”[Mesh]).

Two authors (MG and MR) searched databases. If there was a disagreement, a consensus was reached following discussion. Details about population, intervention, comparator, and outcome (PICO) are described in Table 1.

**TABLE 1:** Detailed information about population, intervention, comparator, and the outcome (PICO)

PICO items	Definition
Population	Diabetic patients (Type I or Type II)
Intervention	Low‐sodium diet
Comparison	High‐sodium diet or Sodium supplementation
Outcome	Systolic blood pressure, Diastolic blood pressure, Mean arterial pressure

### 2.2 Inclusion and exclusion criteria

Clinical trials in which a low‐sodium diet (LSD) compared with a high‐sodium diet (HSD), or sodium supplementation, in diabetic patients (type I or type II diabetes) were included. We excluded the following studies: (1) animal studies, (2) studies that assessed the effect of a medication without any intervention in dietary sodium intake, (3) studies that prescribed dietary approach to stop hypertension (DASH), (4) observational studies, and (5) review studies.

### 2.3 Data extraction

The following information was collected from each study: the name of authors, the year of publication, sex and mean age of participants, design of the study, type of diabetes, amount of dietary sodium in both intervention and control groups, the method for assessing diet compliance, duration of intervention, reported data for blood pressure including SBP, DBP, and mean arterial pressure (MAP), and the health status of participants. Almost all of the studies used the urine analysis method to ensure the compliance of the diet.

### 2.4 Quality assessment

Two authors (MG and MR) evaluated the quality of the articles based on Cochrane Collaboration's tool (Higgins et al., 2011), including: (1) random sequence generation, (2) allocation concealment, (3) blinding of participants and personnel, (4) blinding of outcome assessment, (5) incomplete outcome data, (6) selective reporting, and (7) other sources of bias. Results were divided in three groups: low risk of bias, high risk of bias, and unclear risk of bias. The interpretation of the quality assessment results based on guidelines was as follows: good (low risk for more than 2 items), fair (low risk for 2 items), or weak (low risk for less than 2 items; Higgins et al., 2011).

### 2.5 Statistical analysis

Effect size was calculated using mean and standard deviation (SD) of SBP, DBP, and MAP in the low‐sodium and high‐sodium groups. Standard errors (SE) were converted to SD using the formula SD = SE × √N. In studies that reported 95% confidence interval (CI), SD was calculated using the formula SD = √N × (upper limit − lower limit) ÷ 3.92 (Deeks et al., 2019). A random‐effects model was applied to calculate overall effect size for each outcome (Deeks et al., 2019). All data were reported in the same unit through the studies. Therefore, we reported overall effect sizes in the form of weighted mean difference (WMD). The heterogeneity between included studies was evaluated using *I* ^2^ statistics (Deeks et al., 2019). When a significant between‐study heterogeneity was observed, we performed pre‐planned subgroup analyses based on study design (cross‐over or parallel), study duration (≤1 week or >1 weeks), and health condition of patients (hypertensive or normotensive) to detect possible sources of heterogeneity. Between‐subgroup heterogeneity was determined using a fixed‐effects model (Deeks et al., 2019). We tested the robustness of the overall effect sizes using sensitivity analysis, whilst Begg's rank correlation test (Begg, 1994), as well as Egger's linear regression test (Egger et al., 1997), were applied to detect publication bias. All statistical analyses were performed using Stata software (version 11.2; Stata Corporation, College Station, TX, USA). Analyses were two‐tailed, and statistical significance was set at *p* < .05, a priori.

## 3 RESULTS

### 3.1 Literature search result

**FIGURE 1:**
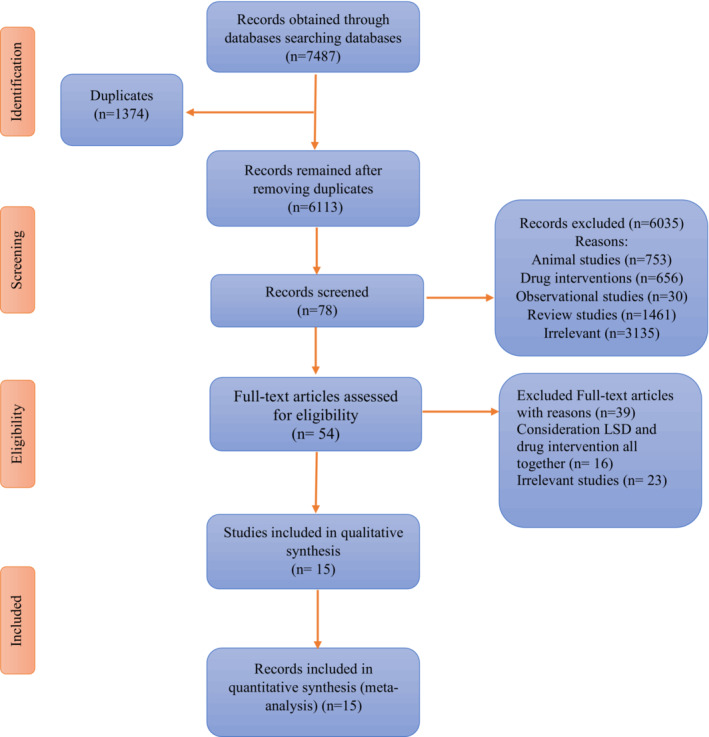
The process of database searches and study selection.

The process of database searches and study selection are shown in Figure 1. A total of 7484 articles were obtained in the basic search (3227 articles from PubMed database, 794 publications from ISI Web of Science, and 3466 results from Scopus database center). Duplicates (*n* = 1374) were excluded, and the titles and abstracts of the remaining articles were reviewed based on inclusion and exclusion criteria, and irrelevant articles were subsequently excluded. Finally, the full‐texts of 78 articles that were apparently appropriate were reviewed, and, finally, 15 articles were retained.

### 3.2 Study characteristics

Details of the included studies are detailed in Table 2. Ten studies were conducted in European countries (Parvanova et al., 2018; de Faria et al., 1997; Dodson et al., 1989; Gerdts et al., 1996; Lambert et al., 1997; Muhlhauser et al., 1996; Suckling et al., 2016; Trevisan et al., 1998; Vedovato et al., 2004; Wenstedt et al., 2020), two in the United States (Olshan et al., 1982; Tuck et al., 1990), and three in Japan (Imanishi et al., 2001; Iuchi et al., 2016; Yokokawa et al., 2020), and the publication date ranged from 1982 to 2021. The mean age of participants ranged from 24 to 66 years old, and the design of most studies was cross‐over, except for three studies that used a parallel design (Imanishi et al., 2001; Muhlhauser et al., 1996; Yokokawa et al., 2020). The sample size ranged from 10 to 753 and most studies recruited both genders, except for two studies which enrolled men only (Olshan et al., 1982; Wenstedt et al., 2020). The amount of sodium in low sodium diets ranged from 20 to 104 mmol/day (460 to 2400 mg/day) and from 80 to 300 mmol/day (1800 to 6900 mg/day) in high sodium diets. All studies had an intervention that increased or decreased the dietary sodium, with or without sodium supplementation, although participants in one study only received nutritional recommendation to reduce sodium intake (Yokokawa et al., 2020). Five studies reported MAP (de Faria et al., 1997; Olshan et al., 1982; Trevisan et al., 1998; Tuck et al., 1990; Vedovato et al., 2004), two studies measured MAP, SBP, and DBP (Gerdts et al., 1996; Wenstedt et al., 2020), and the other studies reported SBP and DBP.

**TABLE 2:** Characteristics of included studies to systematic review

Author (year)	Sample size (gender)	Mean age (years)	Design	Type of diabetes	Sodium intake in intervention group	Sodium intake in control group	Compliance	Duration (days)	Reported data	Results	Notes about subjects
Olshan (1982)	43 (male)	52	Cross‐over	Type 2	140 mEq /24 h	30 mEq/24 h	24‐h urine collection	6	MAP	No significant changes	Hypertensive diabetic subjects
Dodson (1989)	34 (23M:11F)	61.5	Cross‐over	Type 2	–	80 mmol/day	24‐h urine collection	30	SBP DBP	LSD reduced SBP (not DBP)	Mild hypertensive diabetic patiens
TUCK (1990)	26 (both: ND)	55	Cross‐over	Type 2	250‐mEq	20‐mEq	24‐h urine collection	6	MAP	HSD increased MAP	Normotensive and hypertensive diabetic patients
GERDTS (1996)	30 (21M:9F)	46	Cross‐over	Type 1	50 mmol	153 mmol	24‐h urine collection	6	SBP DBP	LSD reduced SBP and DBP in SSS	Hypertensive diabetic subjects
Miihlhauser (1996)	16 (12M:4F)	32	Parallel	Type 1	90 mmol/day	190 mmol/day	24‐h urine collection	28	SDP DBP	No significant changes	IDDM patients with increased proteinuria
de Faria (1997)	10 (7M:3F)	30	Cross‐over	Type 1	100 mmol/day	300 mmol/day	24‐h urine collection	7	MAP	No significant changes	Normotensive diabetic subjects
Lambert (1997)	64 (24M:40F)	24.9	Cross‐over	Type 1	50 mmol/day	200 mmol/day with and without perindopril	24‐h urine collection	5–8	SBP DBP	HSD increased SBP and DBP	Normotensive diabetic subjects
Trevisan (1998)	16 (12M:4F)	40	Cross‐over	Type 1	25 mmol	250 mmol	24‐h urine collection	6	MAP	HSD increased MAP	Normotensive and hypertensive diabetic patients
Imanishi (2001)	32 (19M:13F)	61	Parallel	Type 2	80 mmol/day	200 mmol/day	24‐h urine collection	7	SBP DBP	HSD increased SBP in both normo and micro albuminuric and DBP in microalbuminuric	Normotensive micro–macro‐normo abuminuric diabetic patients
Vedovato (2004)	41 (31M:10F)	58.5	Cross‐over	Type2	25 mmol	250 mmol	24‐h urine collection	7	MAP	HSD increased SBP and DBP in microalbuminuric patients but not in normoalbuminuric	hypertensive diabetic patients with microalbuminuria
Iuchi (2016)	10 (7M:3F)	60	Cross‐over	Type 2	<6 g/day		24‐h urine collection	7	SBP DBP	No significant changes	Hypertensive diabetic subjects
Suckling (2016)	46 (24M:22F)	58	Cross‐over	Type 2	<90 mmol/day	180 mmol/day	24‐h urine collection	42	SBP DBP	Clinical and 24 h SBP and 24 h DBP decreased	T2D or impaired glucose tolerance with or without HTN
Parvanova (2018)	115 (102M:13F)	64.4	Cross‐over	Type 2	<100 mEq/day	> 200 mEq/day	24‐h urine collection	90	SBP DBP	SBP and DBP significantly decreased in the LSD but not HSD	Normotensive T2D patients with 24 h albuminuria of more than 300 mg
Wenstedt (2020)	20 (male)	25	Cross‐over	Type 1	<3 g/day	>12gr/day	24‐hr urine collection	8	SBP DBP	HSD increased MAP	Normotensive diabetic subjects
Yokokawa (2020)	753 (386M:367F)	66	Parallel	Type 2	NR	NR	ND	365	SBP DBP	No significant changes	Normotensive and hypertensive diabetic patients

Abbreviations: DBP, diastolic blood pressure; F, female; HSD, High sodium diet; IDDM, Insulin dependent diabetes mellitus; LSD, Low sodium diet; M, male; MAP, mean arterial pressure; ND, not‐defined; NIDDM, non‐insulin dependent diabetes mellitus; NR, Not reported; SBP, systolic blood pressure; SSS, salt sensitive subjects.

### 3.3 Quality assessment

Results of the risk of bias assessment, based on the Cochrane collaboration tool, are showed in Table 3. Nine studies scored 3, and the rest of the studies scored more than 3. Therefore, all the studies were ranked as good quality.

**TABLE 3:** Risk of bias assessment for included randomized controlled clinical trials

Author (year)	Random sequence generation	Allocation concealment	Blinding of participants and personnel	Blinding of outcome assessment	Incomplete outcome data	Selective reporting	Other bias	Score	Overall quality
Olshan (1982)	−	−	−	−	+	+	+	3	Good
Dodson (1989)	+	+	+	+	+	+	+	7	Good
Tuck (1990)	−	−	−	−	+	+	+	3	Good
Gerdts (1996)	−	−	−	−	+	+	+	3	Good
Muhlhauser (1996)	+	+	+	?	+	+	+	6	Good
De faria (1997)	?	?	−	−	+	+	+	3	Good
Lambert (1997)	−	−	−	+	+	+	+	4	Good
Trevisan (1998)	−	−	−	−	+	+	+	3	Good
Imanishi (2001)	?	−	−	−	+	+	+	3	Good
Vedovato (2004)	?	−	−	−	+	+	+	3	Good
Iuchi (2016)	−	−	−	−	+	+	+	3	Good
Suckling (2016)	+	+	+	?	+	+	+	6	Good
Paravona (2018)	+	+	+	?	+	+	+	6	Good
Wenstedt (2020)	?	−	−	−	+	+	+	3	Good
Yokokawa (2021)	+	+	−	−	+	+	+	5	Good

### 3.4 Effect of a low‐sodium diet on SBP

**FIGURE 2:**
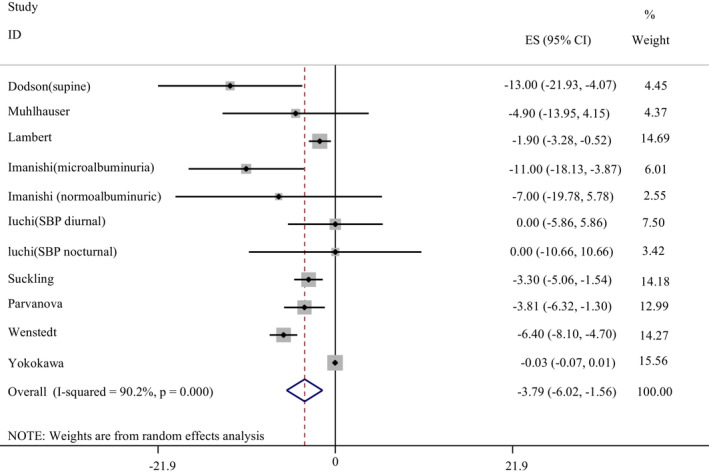
The effect of low‐sodium diet on systolic blood pressure in comparison with a high‐sodium diet.

Eleven studies reported data on the effect of a LSD on SBP compared with a HSD. Pooled analysis showed that a LSD had a significant decreasing effect on SBP in comparison with HSD (WMD: −3.79 mmHg, 95% CI: −6.02, −1.56). Because of a significant between studies heterogeneity (*I* ^2^ = 90.2%, *p* < .001), subgroup analyses were conducted based on duration of intervention, status of the participants, and study design (Table 4). However, none of subgroups could detect the source of heterogeneity. The effect of LSD on overall SBP has been shown in Figure 2.

**TABLE 4:** Results of subgroup analysis

Subgroup	Effect size (*n*)	Pooled effect	*I* ^2^	*p* heterogeneity	*p* between subgroup heterogeneity
SBP (mmHg)					
Design					
Cross‐over	7	−3.79 (−5.82, −1.76)	73.4	.001	.001
Parallel	4	−4.95 (−11.38, 1.47)	73.5	.01	
Health status					
Hypertensive	6	−2.38 (−5.19, 0.42)	77.7	.000	<.001
Normotensive	5	−4.89 (−7.74, −2.04)	80.4	.000	
Duration					
≤1 week	6	−4.20 (−7.49, −0.92)	77.8	.000	<.001
>1 week	5	−3.29 (−6.19, −0.40)	87.1	.000	
DBP (mmHg)					
Health status					
Hypertensive	6	−0.88 (−3.01, 1.25)	65.1	.014	.001
Normotensive	5	−2.23 (−3.46, −1.00)	66.2	.019	
MAP (mmHg)					
Health status					
Hypertensive	5	−5.51 (−13.59, 2.56)	97.2	.000	.0001
Normotensive	5	1.25 (−3.00, 5.49)	93.5	.000	

Abbreviations: DBP, diastolic blood pressure; MAP, mean arterial pressure; SBP, Systolic blood pressure.

### 3.5 Effect of a low‐sodium diet on DBP

**FIGURE 3:**
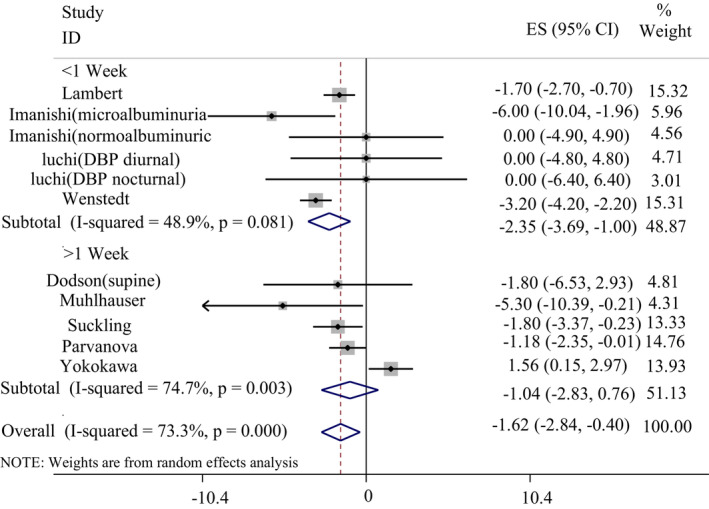
The effect of a low‐sodium diet on diastolic blood pressure in comparison with a high‐sodium diet based on duration of the study.

**FIGURE 4:**
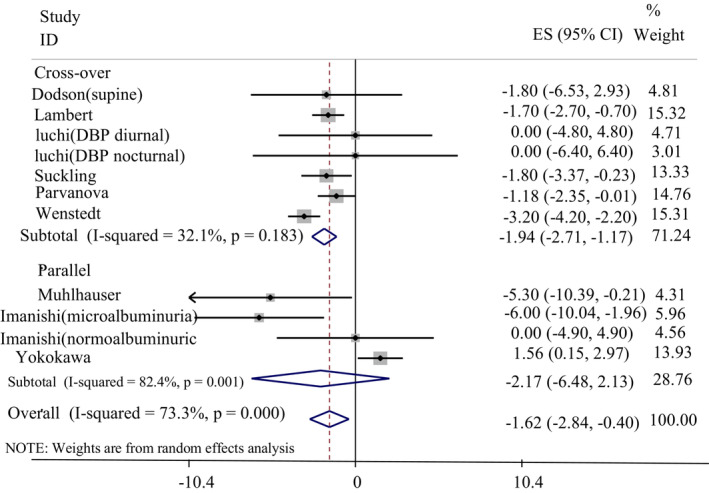
The effect of a low‐sodium diet on diastolic blood pressure in comparison with a high‐sodium diet based on the study design.

Ten studies reported data on the effect of LSD on DBP compared with a HSD. Pooled analysis showed a significant decrease in DBP after consuming a LSD compared with HSD (WMD: −1.62 mmHg, 95% CI: −2.84, −0.40). Because of a significant between study heterogeneity (*I* ^2^ = 73.3%, *p* < .001), a subgroup analysis was conducted based on duration of study, study design, and the health status of the participants. Subgroups based on duration (≤1 week: WMD: −2.35 mmHg, 95%CI: −3.69, −1.00, *I* ^2^ = 48.9%, *p* = .081, >1 week: WMD: −1.04 mmHg, 95% CI: −2.83, 0.76, *I* ^2^ = 74.7%, *p* = .003) and design (cross‐over: WMD: −1.94 mmHg, 95% CI: −2.71, −1.17, *I* ^2^ = 32.1%, *p* = .183, parallel: WMD: −2.17 mmHg, 95% CI: −6.48, 2.13, *I* ^2^ = 82.4%, *p* = .001) detected significant sources of heterogeneity. The effect of a LSD on DBP based on duration and design subgroup has been shown in Figures 3 and 4, respectively. Subgroup analysis based on the health status of the participants was not significant (Table 4).

### 3.6 Effect of low‐sodium diet on MAP

Pooled analysis showed no significant effect of LSD on MAP compared with HSD (WMD: −1.81 mmHg, 95%CI: −5.49, 1.87). Because of small number of studies conducted on MAP, subgroup analysis was conducted only based on health status, and it could not detect the source of heterogeneity (Table 4).

## 4 DISCUSSION

High blood pressure is one of the important risk factors in increasing cardiovascular diseases (Banach & Aronow, 2012; Collins et al., 1990; Lewington, 2002; MacMahon et al., 1990).Further, people with diabetes tend to concurrently experience higher blood pressure (Gerdts et al., 1996). There are numerous approaches to manage blood pressure in people with diabetes, such as reducing sodium intake, adhering to a DASH diet, or using salt substitutes such as potassium salt.

The current systematic review and meta‐analysis of clinical trials assessed the effectiveness of a LSD compared with a HSD on SBP, DBP, and MAP in diabetic patients. The results identified that a LSD had a significant effect on decreasing both SBP and DBP, but not in MAP. Furthermore, subgroup analyses were conducted based on duration of intervention, status of participants, and study design. Accordingly, the results showed that a LSD significantly reduced DBP in cross‐over study designs and in studies where the duration of interventions was lower than 1 week, as compared to a HSD. Previous investigations reported that higher intake of dietary sodium might be associated with adverse health outcomes including kidney disorders, CVD events, and hypertension (Malta et al., 2018; Mills et al., 2016; Smyth et al., 2016), and reduction in dietary sodium could be considered as a beneficial approach for improvement of health status (Malta et al., 2018).

Based on the results of previous studies, water retention (Abbasnezhad et al., 2020; Mills et al., 2016), impairment activity of sympathetic system (Graudal et al., 2020; Jürgens & Graudal, 2002), endothelial dysfunction (Huang et al., 2020), large arteries stiffness, and oxidative stress (Hooper et al., 2002) following high sodium diets are considered as possible mechanisms underlying the association between HSD and hypertension in salt‐sensitive hypertension (Grillo et al., 2019).

The evidence from previous studies indicates that sodium intake reduction might have a beneficial effect on blood pressure. Indeed, the systematic review and meta‐analysis by Abbasnezhad et al. (2020) illustrated that, among different dietary approaches to decrease blood pressure, reducing dietary sodium was the most efficient dietary modification in SBP reduction in type 2 diabetes. However, no significant effect was observed on DBP, which is inconsistent with our results (Abbasnezhad et al., 2020). Interestingly, the aforementioned review was conducted on normotensive or pre‐hypertensive subjects, however, in our study, both normotensive and hypertensive subjects were included. In addition, the Cochrane reviews of trials evaluated the effect of a LSD on blood pressure compared to a HSD, and revealed that a LSD, in both normotensive and hypertensive subjects, reduced systolic and diastolic blood pressure. Nonetheless, the magnitude of the LSD effect in hypertensive people was greater than normotensive counterparts (Graudal et al., 2020; Jürgens & Graudal, 2002).

A further systematic review and dose–response analysis of randomized trials evaluated the effect of dietary sodium reduction on blood pressure, and the results revealed that a reduction in sodium intake prompted a decrease SBP and DBP, with greater magnitude for non‐white, older, and hypertensive subjects. Also, the shorter duration of the intervention (<15 days) may lead to an underestimation of the effect of LSD on blood pressure (Huang et al., 2020). However, the study by Yokokawa et al. reported that dietary intervention, including reduction in dietary salt, had a positive effect on blood pressure lowering in a short term intervention (6 months), although this significant reduction was eliminated at 12 months (Yokokawa et al., 2020). In addition, the study by Hooper et al. (2002) observed that the effectiveness of LSD on reduction of urinary excretion of sodium, and subsequently blood pressure, in short term trials was greater than longer duration trials, which is concordant with our findings. It is evident that the compliance and maintenance of LSD in long term trials is difficult, and adherence appears to attenuate over time. In addition, the number of studies that evaluated the effect of LSD on blood pressure in interventions with longer durations were limited, and therefore may have impacted our ability to accurately consider the effect of longer‐term interventions.

The result of current subgroup analysis demonstrated that the reduction in SBP and DBP following LSD intervention was significant in studies with a cross‐over design, but not in parallel design. Indeed, this suggests that study design may be a key driver in attaining accurate and reliable results (Dodson et al., 1989; Wenstedt et al., 2020; Yokokawa et al., 2020).

The Na/K urine excretion is one of the indices for assessing the blood sodium load. Studies have shown that urinary potassium excretion is inversely related to blood pressure, and potassium supplementation reduces blood pressure. The effect of a high Na/K on blood pressure is greater than the effect of each one independently, and a higher ratio is associated with a higher risk of cardiovascular disease (Tabara et al., 2015). Therefore, future studies should be focused on this variable rather than sodium or potassium separately.

Many studies have been conducted to assess the effect of salt substitutes, such as potassium chloride, sodium malate, and monosodium glutamate, on blood pressure. The results of a meta‐analysis conducted on six clinical trials (using sodium‐magnesium enriched salt) demonstrated that the use of salt substitutes significantly reduces SBP. This reduction was less in the case of DBP (Peng et al., 2014); nevertheless, using potassium chloride as a salt substitute may have cause hyperkalemia in patients with insufficient renal function, and therefore, salt substitutes should be used with caution.

The current meta‐analysis has several strengths, for instance, a comprehensive literature search was conducted to detect trials that assessed the effect of LSD on hypertension in diabetic patients. In addition, to find the source of heterogeneity, a pre‐defined subgroup analysis was performed based on potential confounders, including study design, duration of study, and health status of patients. However, some limitations should be noted. First, a relatively small number of studies were included in this meta‐analysis. Second, most of included studies did not report the effect of LSD on hypertension in males and females, separately, thereby precluding any sex‐based differentiation. Third, the pre‐defined subgroup analysis could not completely eliminate between‐study heterogeneity. Clearly, the above limitations should be addressed in future studies.

## 5 CONCLUSION

In conclusion, LSD had a beneficial effect on SBP and DBP reduction in diabetic patients. However, no significant effect was found in MAP. In addition, the results showed that LSD significantly reduced DBP in cross‐over study designs and when duration of interventions was lower than 1 week, as compared to HSD. To verify our findings and elucidate the underlying mechanisms, further research with different study designs and intervention durations are required.

## CONFLICT OF INTEREST

Authors had no conflict of interests.

## ETHICAL APPROVAL

Not applicable.

## Data Availability

The data that support the findings of this study are available from the corresponding author upon reasonable request.
